# Development and validation of an HPLC method for the determination of vancomycin in human plasma and its comparison with an immunoassay (PETINIA)

**DOI:** 10.1186/s40064-016-1778-4

**Published:** 2016-02-18

**Authors:** Muhammad Usman, Georg Hempel

**Affiliations:** Department of Pharmaceutical and Medicinal Chemistry - Clinical Pharmacy, University of Muenster, Corrensstrasse 48, 48149 Muenster, Germany

**Keywords:** HPLC, PETINIA (Siemens), TDM, Validation, Vancomycin

## Abstract

Vancomycin (VAN) is among those antibiotics for which therapeutic drug monitoring is highly recommended. For this purpose a reliable method with small sample volume was required for quantification of VAN in human plasma. Therefore, a selective and sensitive method of high performance liquid chromatography was developed and validated. The separation was carried out isocratically by using a mobile phase NH_4_H_2_PO_4_ (50 mM, pH 2.2)–acetonitrile (88:12, v/v) at a flow rate of 0.36 mL/min on a nucleodur C18 column (125 mm × 4.6 mm, 5 µm) with UV detection at 205 nm. Sample preparation was done by deproteination of plasma with 70 % perchloric acid and a liquid/liquid extraction. Validation was performed according to the European Medicines Agency guideline. The method showed linearity over the range of 0.25–60 mg/L with a coefficient of determination r^2^ ≥ 0.999 and a lower limit of quantification of 0.25 mg/L. No interference was observed in blank plasma samples at the retention time of VAN. The percentage relative recovery and coefficient of variation (CV%) values for accuracy and precision were within the acceptable limits. Stability was proved at room temperature for 24 h, after repeated freeze and thaw cycles and storage at −20 °C for 3 months. A good correlation was observed (r = 0.947) by comparing with the results of an immunoassay (PETINIA, Siemens) in 289 samples. In conclusion the method proved simple, sensitive and cost effective for quantification of VAN in human plasma.

## Background

Vancomycin (VAN), a glycopeptide antibiotic is used against infections caused by Gram-positive bacteria, particularly methicillin-resistant *Staphylococcus aureus* (MRSA) (Van Bambeke 2004; Lundstrom and Sobel 2004; Wilhelm and Estes 1999; Kullar et al. 2011). Under-dosing of VAN may lead to insufficient eradication of the bacteria and over-dosing is associated with toxicity (Ingram et al. 2008). Therapeutic drug monitoring (TDM) is highly recommended for optimizing VAN therapy (Martin et al. 2010). TDM involves the measurement of drug concentrations in plasma, serum or blood in order to individualise dosage for maintaining the drug concentrations within a target range (Kang and Lee 2009). The recommended target trough concentration range (TTCR) of vancomycin is 10–15 mg/L (Helgason et al. 2008) but for more resistant strains of MRSA 15–20 mg/L is recommended by British National Formulary (BNF) (Joint Formulary Committee 2015).

Many methods for the quantification of VAN in biological fluids have been developed and validated. These include radio immunoassay (RIA) (Ackerman et al. 1983), enzyme multiplied immunoassay (EMIT) (Yeo et al. 1989), fluorescence polarization immunoassay (FPIA) (Ackerman et al. 1983; Filburn et al. 1983) and HPLC methods by using either UV detection (Hagihara et al. 2013; Jesus Valle et al. 2008; Diana et al. 2003; Ye et al. 2008; Farin et al. 1998; Plock et al. 2005; Hoagland et al. 1984), diode array detection (Backes et al. 1998; Cao et al. 2013; Hu et al. 2012) or fluorescence detection (Abu-Shandi 2009). A more sensitive but expensive LC–MS/MS method has also been recently developed and validated with limit of quantification of 0.3 mg/L (Oyaert et al. 2015).

These techniques have been compared with one another. FPIA was compared with RIA and both techniques were proved comparable with correlation coefficient of r = 0.99 (Ackerman et al. 1983). The values obtained from EMIT were compared with FPIA and a linear relationship was observed. However, EMIT lost precision at concentrations above 30 mg/L (Yeo et al. 1989). Overestimation of VAN was observed by FPIA in samples obtained from peritoneal dialysis patients when compared with results of HPLC. The authors concluded that the cross-reactivity due to the degradation products of VAN was the reason for this overestimation (Morse et al. 1987). A good correlation was observed between the results of RIA and HPLC (Hagihara et al. 2013) and between FPIA and HPLC (Ristuccia et al. 1984; Farin et al. 1998). Recently, a very low concentration (<4 mg/L) was observed with the Beckman Coulter PETINIA method when compared with PETINIA (Siemens) (57.7 mg/L) and FPIA (71.2 mg/L). Interference with IgM was concluded as a reason by the authors (Gunther et al. 2013).

Immunoassay techniques are widely used in clinical practice because of their speed and simplicity. These methods are effective within the therapeutic concentration range (5–20 mg/L) with quantification limit of 5 and 2 mg/L, respectively for EMIT and FPIA (Sym et al. 2001). However, when low levels of VAN are expected, only more sensitive HPLC method is suitable (Farin et al. 1998). Higher concentrations of VAN are also of interest when developing new dosing regimens and these high concentrations are not reliably quantified by the immunological methods (Yeo et al. 1989). Immunoassays used in clinical practice have the disadvantage of cross-reactivity with related substances such as metabolites of VAN (Morse et al. 1987). Therefore, HPLC can be a better analytical approach for quantification of VAN in biological fluids in order to determine the pharmacokinetics of VAN particularly when very small concentrations are expected. A recent HPLC method for quantification of VAN in human plasma has been validated with lower limit of quantification (LLOQ) of 1 mg/L (Hagihara et al. 2013). A high flow rate was used in this method (1.2 mL/min) which ultimately increases the cost of analysis. A more sensitive and cost effective HPLC method with comparable retention time was still required.

The purpose of this investigation is to develop and validate a reliable, sensitive, selective and cost-effective HPLC method for the quantification of VAN in human plasma using a small sample volume in order to determine pharmacokinetics of VAN in elderly or paediatric patients. This investigation also aims to show how the results of this HPLC method compare with the results obtained by using PETINIA (Siemens) in a series of samples already obtained from patients and analysed for TDM.

## Methods

### Chemicals and reagents

VAN and ammonium di-hydrogen phosphate were purchased from Sigma-Aldrich Chemie GmbH. Acetonitrile was HPLC grade and all other reagents were analytical grade (ethyl acetate, perchloric acid, phosphoric acid etc.). The citrated plasma from healthy donors was supplied by the Department of Transfusion Medicine, University Hospital Muenster (UKM) Germany. Double distilled water was prepared in laboratory using Milli-Q^®^ direct water purification system.

Serum samples from patients receiving VAN were supplied by the Central laboratory of the UKM, Germany. The samples from patients with suspected or documented infections with VAN-sensitive bacteria were drawn as part of their clinical routine in order to monitor the VAN concentrations.

### Sample preparation

Stock solution (1 g/L) of VAN was prepared in double distilled water and a working solution (120 mg/L) was prepared in plasma. Calibration standard solutions and quality control (QC) samples were prepared by serial dilution with plasma. The final concentrations for calibration standard solutions were 60, 30, 10, 1, 0.5 and 0.25 mg/L and for QC samples were 50, 25, 0.5 and 0.25 mg/L as higher quality control (HQC), medium quality control (MQC), lower quality control (LQC) and LLOQ respectively.

### Extraction

VAN was extracted by deproteination of 0.2 mL plasma sample with 10 µL of 70 % perchloric acid. The mixture was vortex-mixed for 1 min and centrifuged at 10,500*g* for 10 min. The supernatant was transferred to another polypropylene tube and washed with 1 mL of ethyl acetate by mixing for 1 min and centrifugation at 400*g* for 2 min. The supernatant layer of ethyl acetate was discarded and an aliquot of 40 µL was injected to HPLC for analysis.

### HPLC conditions

The HPLC system comprised of a LC-20AT pump, SIL-10AD auto injector and the UV detector SPD-10A (Shimadzu Germany, Langenfeld). A mixture of 50 mM NH_4_H_2_PO_4_ adjusted to a pH of 2.2 and acetonitrile (88:12, v/v) was used as the mobile phase. The pH was adjusted using phosphoric acid. Separation was carried out isocratically with a flow rate of 0.36 mL/min on a nucleodur C18 column (125 mm × 4.6 mm, 5 µm, Macherey–Nagel) at room temperature with UV detection at 205 nm.

### Validation

The European Medicines Agency (EMA) guideline (European Medicines Agency 2011) was followed for validation of the method. Selectivity, linearity, LLOQ, accuracy, precision (within run and between run) and stability were assessed during method validation.

### Selectivity

Selectivity was observed by comparing the chromatograms of spiked and drug free plasma samples. For this purpose spiked sample of VAN (60 mg/L) and blank plasma samples from six different sources were prepared and injected. Selectivity was particularly investigated for Meropenem and Imipenem, which are frequently co-administered with VAN.

### Linearity and sensitivity

Calibration standard solutions were prepared in plasma from working solution (120 mg/L). Five calibration curves ranging from 0.25 to 60 mg/L were run to establish linearity by using weighted linear regression analysis. The calibration graph was created by plotting VAN concentrations versus the corresponding peak heights. Linearity was observed in term of coefficient of determination (r^2^). The concentration of VAN in each calibration standard was back-calculated using calibration curve and the percentage relative recovery and CV% were determined. The LLOQ defined by EMA guidelines is the lowest concentration that can be quantified with an acceptable accuracy and precision i.e. <20 %.

### Accuracy and precision

Quality control samples (n = 5) were prepared at four different levels HQC, MQC, LQC and LLOQ and analysed thereafter to evaluate with-in run accuracy and precision. The concentrations of VAN were calculated from a standard calibration curve that was simultaneously obtained. To estimate between run accuracy and precision, each level of quality control was analysed on five different days and concentrations were calculated using calibration curves obtained on the same days. Accuracy was estimated at each level by comparing observed concentration with the nominal concentration as a mean percentage relative recovery, while precision was observed in terms of CV%.

### Stability

Stability of VAN in plasma was observed at HQC and LQC levels. To evaluate stability at room temperature, five replicates of both levels were prepared in plasma and stored at room temperature for 24 h. Freeze and thaw stability was observed after four freeze and thaw cycles. The samples were frozen after each cycle for at least 24 h before thawing. For long term stability HQC and LQC (n = 5) were prepared and stored at −20 °C for 3 months. The concentration of VAN in all samples was determined through freshly prepared calibration curves.

### Comparison with PETINIA

The particle enhanced turbidimetric inhibition immunoassay (PETINIA) technique uses a synthetic particle-vancomycin conjugate and a monoclonal VAN specific antibody. VAN present in sample compete with VAN on the particles for available antibody and decreases the rate of aggregation. Therefore, the rate of aggregation is inversely proportional to the concentration of VAN in the sample. The rate of aggregation is measured using bichromatic turbidimetric readings at 340 and 700 nm. The upper limit of quantification was 50 mg/L and the lowest quantified level of VAN reported by PETINIA analysis was 2 mg/L.

A Bland–Altman plot (Bland and Altman 1999) of 289 samples analysed by HPLC and PETINIA (Siemens) was constructed between mean values of HPLC and PETINIA and the percentage differences between the results of both methods. Bias was estimated as mean of percentage differences between both methods. The upper and lower limits of agreement were plotted with 95 % confidence interval as (Limit of agreement = Mean ± 1.96 × SD). Pearson correlation analysis was also performed by comparing the results of HPLC and PETINIA (Siemens). The ethical approval was not required because routine laboratory samples were used which were already obtained and analysed for TDM. The identification of the patients (Name, date of birth and registration number) were removed from the samples before analysis. Although, the method is developed and validated in plasma, a negligible interference has been observed when comparing the matrix effect between human plasma and mouse serum on VAN analysis (Hagihara et al. 2013). Both matrices (plasma and serum) have also been proved comparable in another recent analytical study (Montenarh et al. 2014).

## Results

### Selectivity

**Fig. 1:**
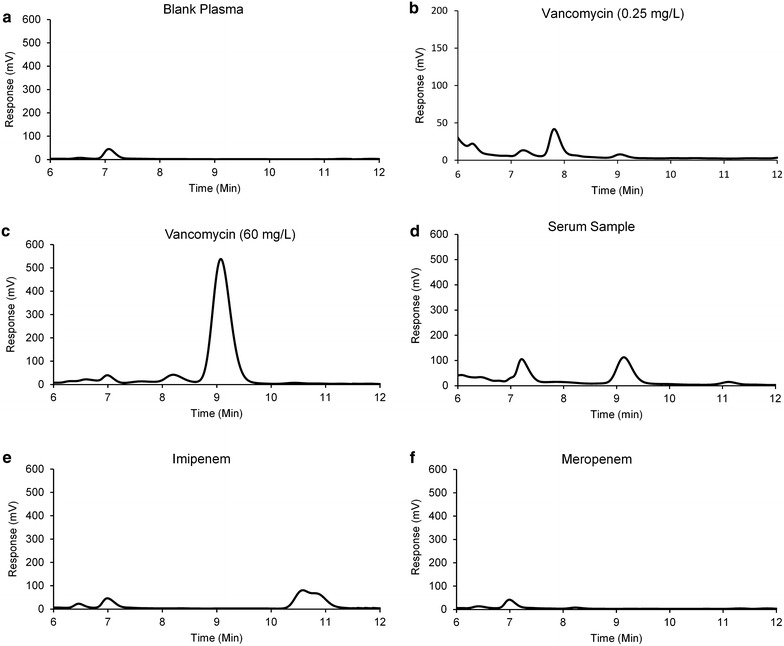
Chromatograms of blank plasma (**a**), plasma spiked with vancomycin 0.25 mg/L (**b**) and 60 mg/L (**c**), a patient’s sample 14.2 mg/L (**d**), plasma spiked with imipenem 60 mg/L (**e**) and plasma spiked with meropenem 60 mg/L (**f**)

VAN was eluted at 9.1 min and no interfering peak was observed at this time when comparing the chromatogram of VAN 60 mg/L with meropenem 60 mg/L, imipenem 60 mg/L and drug free plasma samples (Fig. 1). Therefore, the method can be considered as selective for VAN with no interference with components of plasma and frequently co-administered antibiotics.

### Linearity and sensitivity

The calibration curves (n = 5) were linear with r^2^ ≥ 0.999 over the range of 0.25–60 mg/L. The mean ± SD value for slope was 8231.2 ± 187.7 and the intercept was 2406.7 ± 963.7. The back calculated concentrations of calibration standards are shown in Table 1. The lowest level in the calibration curve (0.25 mg/L) is the LLOQ with percentage relative recovery 100.3 % and a CV 3.23 %.

**Table 1:** Back calculated concentrations of calibration standards

Back calculation (n = 5)	Nominal concentration (mg/L)					
	60	30	10	1	0.5	0.25
Mean (mg/L)	58.0	30.2	10.3	0.99	0.49	0.25
SD	2.09	1.24	0.31	0.07	0.02	0.01
Recovery (%)	96.6	100.7	103.5	99.2	99.6	100.3
CV (%)	3.61	4.11	3.03	6.93	4.61	3.23

### Accuracy and precision

The results for within run and between run accuracy and precision are given in Table 2. The mean percentage relative recovery (n = 5) of VAN for within run accuracy was between 93.1 and 115.0 % while CV ≤ 12.2 %. The values of mean percentage relative recovery for between run accuracy and precision were between 91.5 and 101.2 % whereas CV ≤ 17.8 %.

**Table 2:** Accuracy and precision

Accuracy and precision (n = 5)	Nominal concentration (mg/L)			
	50 (HQC)	25 (MQC)	0.5 (LQC)	0.25 (LLOQ)
*Within run* ^a^				
Mean (mg/L)	53.3	23.3	0.5	0.29
SD	3.20	1.02	0.06	0.01
Recovery (%)	106.6	93.1	99.5	115.0
CV (%)	6.0	4.40	12.2	3.03
*Between run* ^b^				
Mean (mg/L)	50.6	24.9	0.46	0.24
SD	3.87	2.0	0.06	0.04
Recovery (%)	101.2	99.7	91.5	97.9
CV (%)	7.64	8.03	13.5	17.8

^a^Analysed on same day

^b^Analysed on five different days

### Stability

The results for the stability tests are given in Table 3. VAN proved stable at room temperature for 24 h with mean percentage relative recovery (n = 5) for LQC and HQC as 102.6 and 96.9 %, respectively and a CV ≤ 3.32 %. After four freeze and thaw cycles, the mean percentage recoveries were 96.8 and 108.2 % respectively and a CV ≤ 11.4 %. For long-term stability VAN also proved stable with mean percentage relative recovery (n = 5) of 87.45 and 91.67 % respectively for LQC and HQC while CV ≤ 4.94 %.

**Table 3:** Stability

Stability (n = 5)	Nominal concentration (mg/L)	
	50 (HQC)	0.5 (LQC)
*Short term stability* ^a^		
Mean (mg/L)	48.5	0.51
SD	1.18	0.02
Recovery (%)	96.9	102.6
CV (%)	2.43	3.32
*Freeze–thaw stability* ^b^		
Mean (mg/L)	50.6	0.48
SD	4.73	0.06
Recovery (%)	108.2	96.8
CV (%)	8.74	11.4
*Long term stability* ^c^		
Mean (mg/L)	45.8	0.44
SD	0.8	0.02
Recovery (%)	91.7	87.5
CV (%)	1.75	4.94

^a^After storage at room temperature for 24 h

^b^After four freeze and thaw cycles

^c^After storage at −20 °C for 3 months

### Comparison with PETINIA

**Fig. 2:**
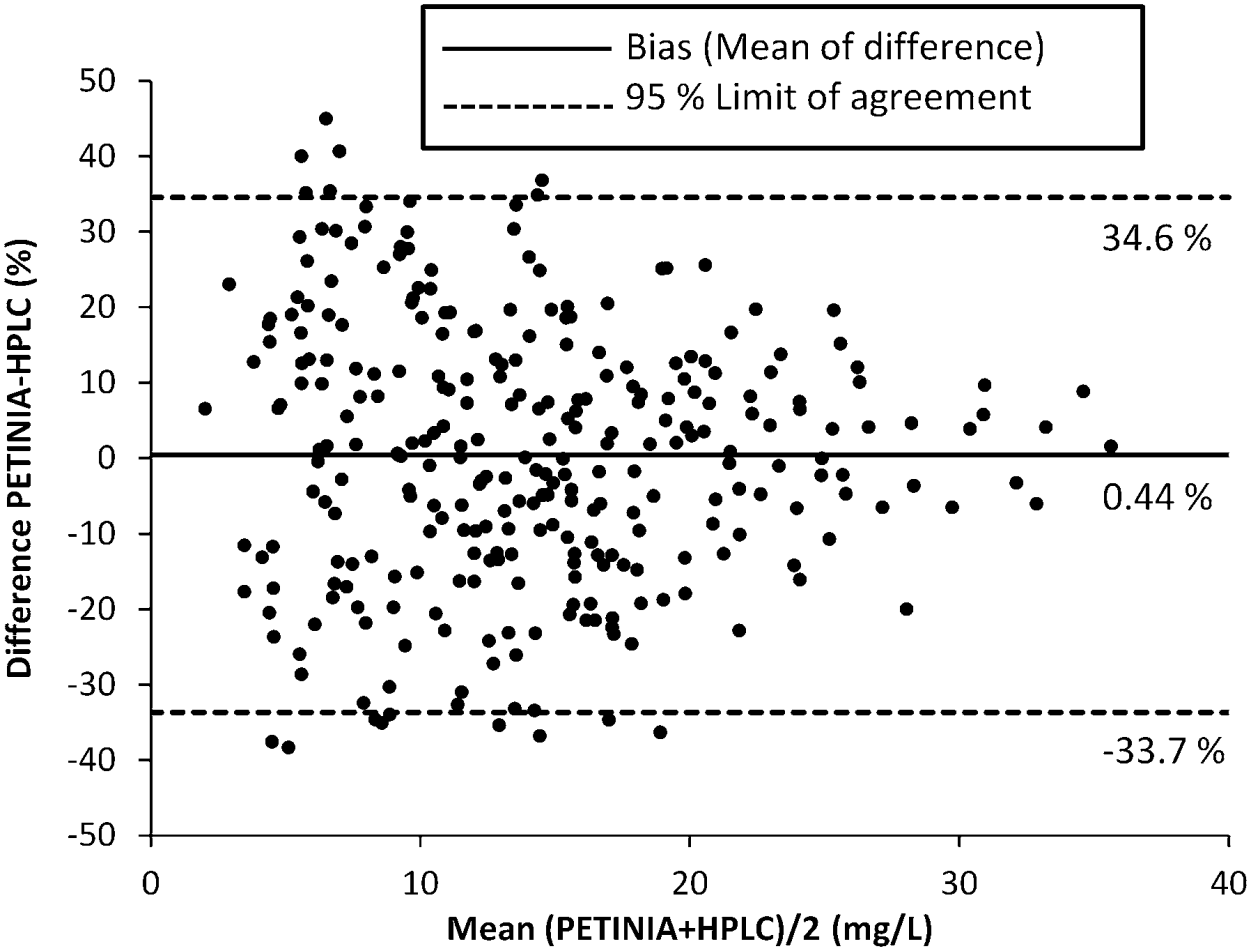
Bland–Altman plot of differences in 289 samples analysed by HPLC and PETINIA (Siemens)

**Fig. 3:**
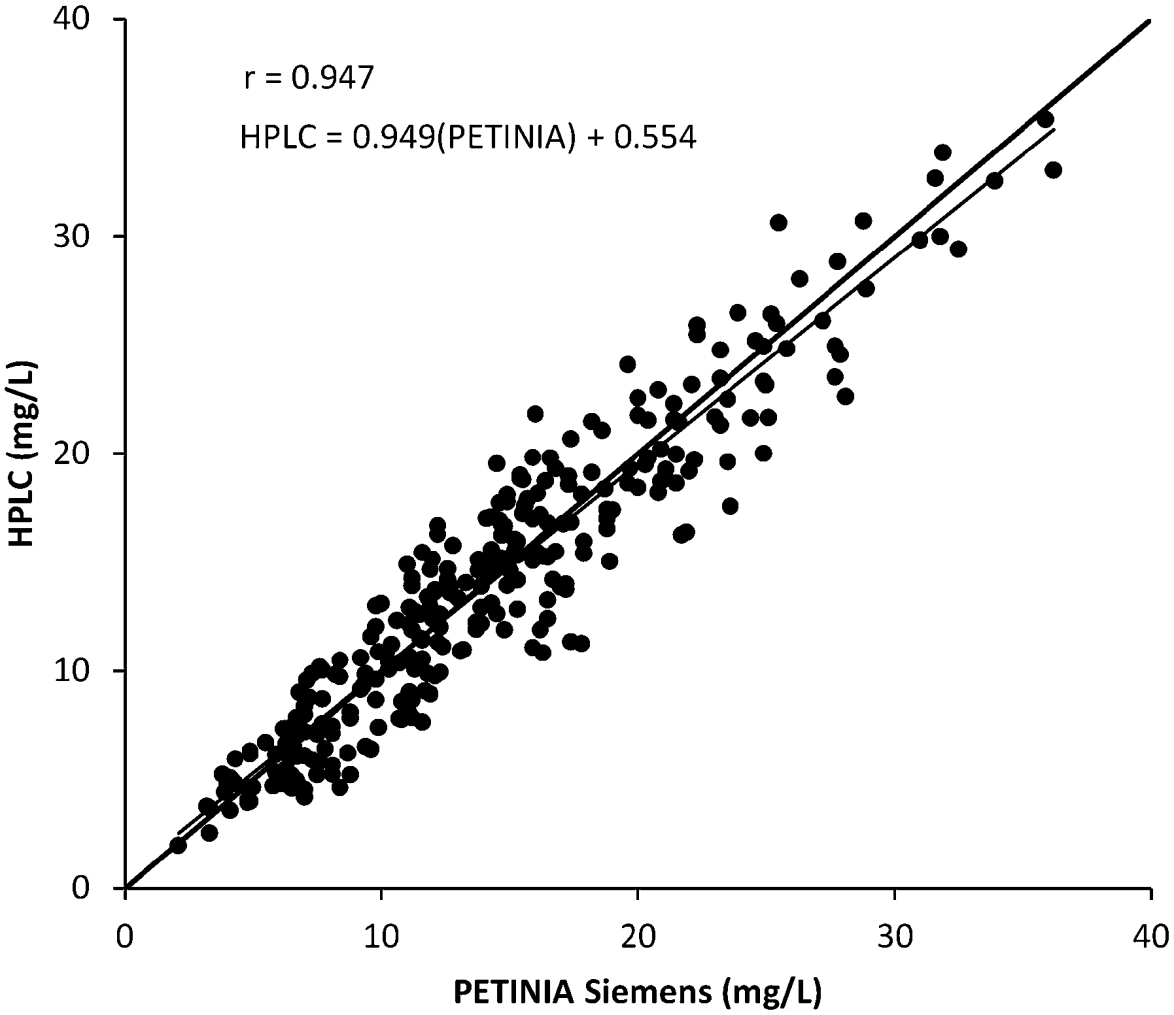
Pearson correlation analysis of VAN concentrations in 289 samples analysed by HPLC and PETINIA (Siemens) showing a good agreement between results produced by both methods

The comparison of this HPLC method was made with PETINIA in 289 samples out of which 148 (51 %) samples were within the recommended TTCR (10–20 mg/L) while the number of samples below and above the TTCR were 92 (32 %) and 49 (17 %) respectively. The Bland–Altman plot for comparison of this method with PETINIA in 289 samples is shown in Fig. 2. The mean of difference was found as 0.44 and 95 % limit of agreement ranges from −33.4 to 34.6 %. The Pearson correlation analysis is shown in Fig. 3 with a correlation r = 0.947 between the results of HPLC and PETINIA (Siemens) in 289 samples. The established equation was: y (HPLC) = 0.949 × (PETINIA) + 0.554.

## Discussion

Different chromatographic conditions including different compositions and pH of mobile phase, different flow rates, different columns, different detectors and wavelengths have been employed for the quantification of VAN in biological fluids (Abu-Shandi 2009; Backes et al. 1998; Diana et al. 2003; Farin et al. 1998; Hagihara et al. 2013; Jesus Valle et al. 2008; Zhang et al. 2007; Cao et al. 2013; Hu et al. 2012; Ye et al. 2008; Plock et al. 2005). Different procedures for extraction of VAN from biological fluids have also been used including solid phase extraction (SPE) (Farin et al. 1998; Zhang et al. 2007; Backes et al. 1998), liquid–liquid extraction (Abu-Shandi 2009; Hagihara et al. 2013; Hu et al. 2012; Plock et al. 2005) and deproteination with perchloric acid (Jesus Valle et al. 2008). In this method the flow rate used (0.36 mL/min) was much lower than 1.2 mL/min (Hagihara et al. 2013) and 1 mL/min (Jesus Valle et al. 2008) while the retention time (9.1 min) was either shorter than 14.4 min (Hagihara et al. 2013) or comparable to 8.5 min (Jesus Valle et al. 2008) reported in other methods which ultimately reduces the cost of analysis. As the method is developed for pharmacokinetic studies of VAN in elderly patients, the volume of plasma sample was kept as low as possible (0.2 mL) in order to reduce the required volume of blood samples from the patients. Extraction was done by the modification of a procedure already in use (Jesus Valle et al. 2008) with the novelty of washing the samples with ethyl acetate after deproteination in order to remove lipophilic interfering components and to enhance the selectivity of method.

This method is four times more sensitive when compared with already developed HPLC method (LLOQ = 1 mg/L) (Hagihara et al. 2013). Another group (Jesus Valle et al. 2008) developed and validated a method with LLOQ 0.1 mg/L in artificial perfusion fluid and lung tissue samples with three calibration ranges (0.1–2, 2–15 and 15–250 mg/L) and a large injection volume (100 µL) which would be difficult to reproduce with plasma samples in clinical situations.

All the results for accuracy and precision were within the limits accepted by EMA guideline (±15 %). The value of CV (17.82 %) in between run accuracy and precision is for LLOQ where the acceptable value should not exceed 20 %.

The stability of VAN in previous studies was observed after storage at room temperature (23 °C) for at least 5 h and after three freeze and thaw cycles (Hagihara et al. 2013), after only freeze and thaw cycles (Jesus Valle et al. 2008) and long term stability after freezing at −70 °C for 2 months (Abu-Shandi 2009). In this study, VAN was proved stable at room temperature for 24 h, after four freeze and thaw cycles and also after freezing for more than 3 months at −20 °C.

In a previous study (Berthoin et al. 2009) only regression analysis was performed for comparison of HPLC and PETINIA in 65 serum samples. We used a substantially large number of 289 samples for the comparison of relatively more sensitive HPLC method with PETINIA by using Bland–Altman analysis and Pearson correlation analysis. The Bland–Altman plot with mean of difference as 0.44 % indicates that both methods are systematically producing similar results. A good correlation (r = 0.947) was also observed with established equation [y (HPLC) = 0.949 × (PETINIA) + 0.554] which demonstrate a good agreement between the results produced by both methods. However, our HPLC method is eight times more sensitive than PETINIA.

## Conclusion

The current HPLC method for the quantification of VAN in human plasma is simple, sensitive and cost effective as the flow rate of mobile phase (0.36 mL/min) is much lower when compared with other pre-existing techniques. The small volume of sample required for analysis also makes it suitable for its intended application to pharmacokinetic studies in elderly or paediatric patients. Moreover this method proved comparable with PETINIA (Siemens) technique which is already used in clinical practice for TDM. However, because of the higher sensitivity and the higher concentration range covered, this HPLC method is preferred when concentrations lower than therapeutic levels or higher levels are to be quantified.
